# Adenoma of the nipple: A clinicopathological report of 13 cases

**DOI:** 10.3892/ol.2014.2000

**Published:** 2014-03-28

**Authors:** MAURIZIO DI BONITO, MONICA CANTILE, FRANCESCA COLLINA, MASSIMILIANO D’AIUTO, GIUSEPPINA LIGUORI, ROSSELLA DE CECIO, GERARDO BOTTI

**Affiliations:** 1 Department of Pathology, National Cancer Institute, Fondazione Pascale Hospital, Naples I-80131, Italy; 2 Department of Senology, National Cancer Institute, Fondazione Pascale Hospital, Naples I-80131, Italy

**Keywords:** nipple adenoma, myoepithelial cells, immunophenotype

## Abstract

Adenoma of the nipple (AN) represents a rare benign mammary proliferation of lactiferous ducts. It appears as an erosive or ulcerative lesion, which in a number of cases is associated with a serous/hematic secretion. AN may be clinically confused with Paget’s disease and histologically with invasive breast carcinoma or breast cancer precursor lesions. Therefore, the histological and immunophenotypic analysis is essential for the differential diagnosis. The present study describes the histopathological characteristics of a first case series of AN.

## Introduction

Adenoma of the nipple (AN) is a rare benign epithelial tumor of the nipple ducts. It generally occurs unilaterally and arises at an average age of between 43–45 years, predominantly in females and rarely in males and adolescents (1–3). The lesion, also known as erosive adenoma and florid papillomatosis, appears similar to a hard-elastic nodule that deforms the nipple, causing swelling or erosion with serous or hematic secretion. AN is often confused with Paget’s disease (4) and the differential diagnosis with breast carcinoma is often difficult (5,6).

AN appears histologically as an extremely heterogeneous tumor entity, particularly due to the following various patterns of growth associated with it: i) ‘sclerosing papillomatosis pattern’, often indistinguishable from sclerosing papilloma; ii) ‘papillomatosis pattern’, florid papillary hyperplasia of ductal epithelium; iii) ‘adenosis pattern’, evident myoepithelial hyperplasia; and iv) ‘mixed proliferative pattern’, combination of three patterns (metaplasia of ducts with cysts, apocrine metaplasia and acanthosis of the epithelium) (7).

A larger series previously reported in the literature referred to a group of 42 American patients and a casuistry of 18 Chinese patients (8,9), collected over several decades, which did not include Italian patients. Only sporadic case reports have been previously described (2,10).

The present study described a series of 13 cases of AN with clinicopathological features, collected within a decade, highlighting the incidence of this benign lesion in the population of Southern Italy. In addition, the requirement of a careful morphological analysis, associated with a relevant immunophenotypic panel, for the recognition of this lesion and differential diagnosis with other breast malignant neoplasms was highlighted.

## Materials and methods

### Clinical information

Cases were selected from the pathological files of the National Cancer Institute, Fondazione Pascale Hospital (Naples, Italy) between January 2003 and April 2013. The World Health Organization (WHO) criteria was strictly applied to establish the diagnosis of AN. Clinical information was recovered from clinical files and a total of 13 cases were identified. All patients signed an informed consent form according to the institutional regulations.

### Immunophenotype analysis

The formalin-fixed, paraffin-embedded (FFPE) tissue block specimens were sectioned (3-μm thick), deparaffinized and rehydrated. Each section was stained with hematoxylin and eosin and then used for immunostaining. Immunohistochemical analyses were performed using an autostainer (BenchMark XT system; Ventana Medical Systems, Inc., Tucson, AZ, USA) according to the manufacturer’s instructions. The following anti-human primary antibodies were used: p63 (Santa Cruz Biotechnology, Inc,. Santa Cruz, CA, USA), caldesmon, calponin, α-smooth muscle actin, CD10, cytokeratin (CK) 5/6 (DakoCytomation, Glostrup, Denmark) and CK8/18 (Novocastra, Newcastle, UK) (Table I).

Stained sections were evaluated by two different pathologists using uniform criteria. Discrepancies were resolved through simultaneous evaluation and discussion of the results. Single-marker expression was recorded as negative/positive and high/low level, following consideration of the expression in reactive surrounding tissue compared with tumoral cells and the specific cut-off of each marker.

## Results

### Clinicopathological features

All AN patients were admitted to the National Cancer Institute, Fondazione Pascale Hospital following a physical examination revealing a well-defined erosive tumor, often serousanguineous, of the breast nipple. Mammography and ultrasonography revealed no mass lesions and calcifications in the two breasts. In total, three cases appeared clinically as Paget’s disease. A total excision of the nipple and areola with an underlying portion of breast tissue was obtained. All clinicopathological parameters of patients are included in Table II.

In summary, all patients were female, with an age range of 20–51 years and an average age of 38 years. The medium size of the lesions was between 0.8 and 1.5 cm.

### Histopathological observations

**Figure 1:**
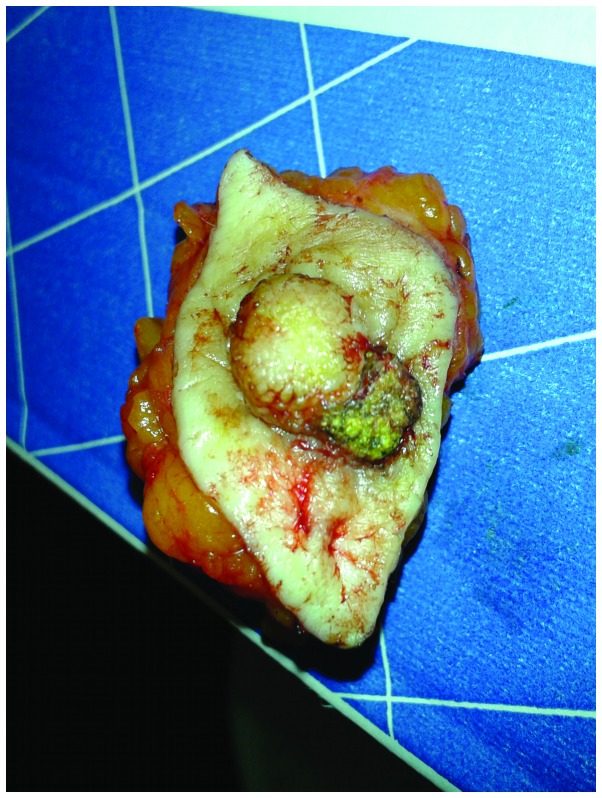
Complete resection of the nipple with erythema, superficial ulceration and crusting.

**Figure 2:**
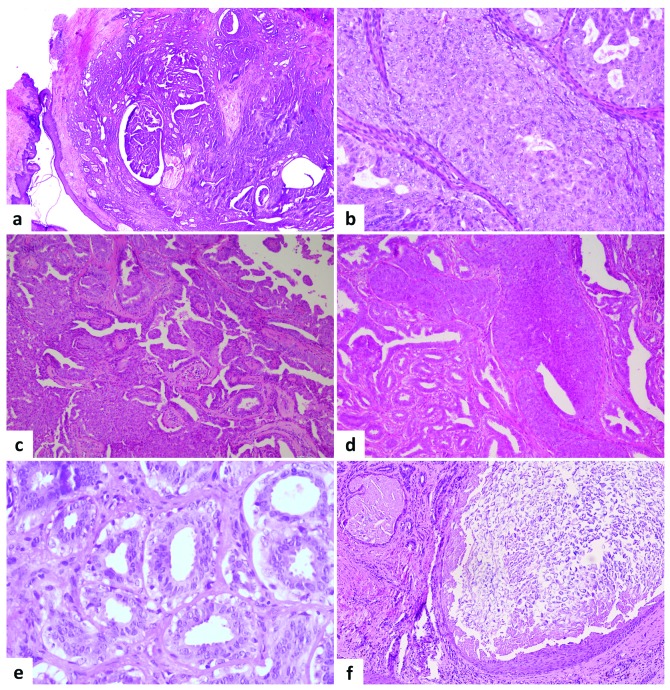
Hematoxylin and eosin morphology. (a) AN lesion overview (magnification, ×10). (b) Papillomatosis pattern with usual ductal hyperplasia (magnification, ×40). (c) Papillomatosis pattern (magnification, ×20). (d) Mixed pattern (papillomatosis and adenosis; magnification, ×20). Adenosis pattern with (e) myoepithelial hyperplasia (magnification, ×40) and (f) keratocysts (magnification, ×20). AN, adenoma of the nipple..

Macroscopically, all lesions presented in the retroareolar region, with no encapsulated nodules and infiltrative margins (Fig. 1). The presence of adenomatous proliferation in the stroma of medium and small caliber ducts, coated by a double layer of cells (epithelial and myoepithelial) was detected in all samples. Only one of the 13 cases appeared with ductal carcinoma *in situ* (DCIS) following the intraoperative examination. The histological features of the 13 lesions were extremely variegated even when the prevalent growth pattern was the papillomatosis pattern with a florid papillary hyperplasia of ductal epithelium. In the majority of cases, the following features were observed: i) presence of fibrosis with distortion of the ducts that may simulate images of pseudo invasion; ii) epithelial hyperplasia with a partial or total obliteration of the lumen; iii) epithelial hyperplasia with intraductal papillary projections; iv) presence of intraductal necrosis; v) presence of cellular monomorphism and/or polymorphism; vi) cellular atypia; and vii) mitosis in 50% of cases. One case showed an adenosis pattern with myoepitelial hyperplasia and two cases showed a mixed proliferative pattern (Fig. 2).

### Immunohistochemical observations

Immunohistochemical studies were performed on all AN specimens. For epithelial cells of the inner layer of ducts, CK8/18 antibodies were used, while myoepithelial cells of the outer layer were highlighted using antibodies against p63, caldesmon, calponin, α-smooth muscle actin, CK5/6 and CD10.

**Figure 3:**
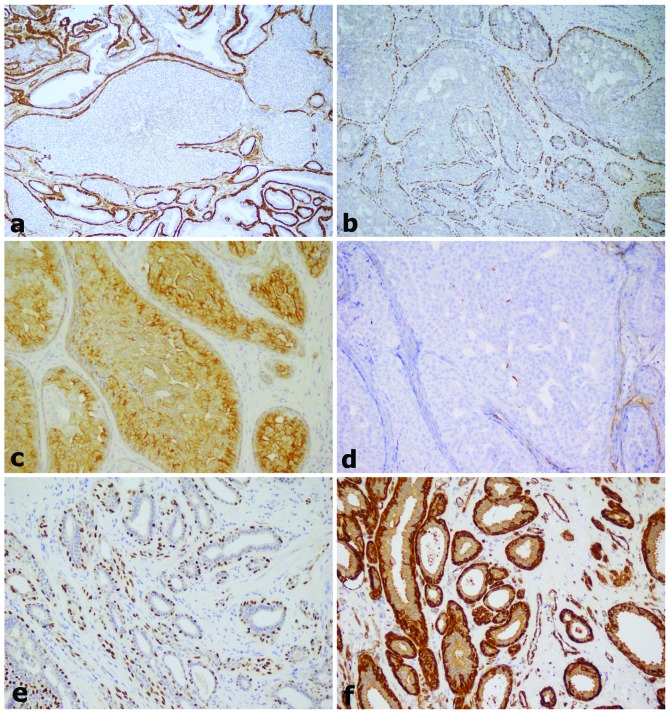
Immunophenotyping. (a) M-actin, (b) p63, (c) cytokeratin 5/6 and (d) cladesmon 1 staining in AN with papillomatosis pattern and (e) p63 and (f) M-actin staining in AN with adenosis pattern (magnification, ×20). M-actin, α-smooth muscle actin; AN, adenoma of the nipple.

The details of positivity/negativity for several markers is included in Table II and shown in Fig. 3.

## Discussion

AN is a rare benign tumor of the breast, which originates from the nipple areola complex generally between the fourth or fifth decade of life. This lesion is almost always unilateral and is often accompanied by a serous/hematic secretion in the nipple. In the WHO classification, AN is defined as ‘a compact proliferation of small tubules lined by epithelial and myoepithelial cells, with or without proliferation of the epithelial component, around the collecting ducts of the nipple’ (11).

However, there is considerable confusion concerning the terms used to define this lesion, due to the diversity of histological pattern with which it occurs. It has been defined as erosive adenomatosis of the nipple, papillary AN, florid adenomatosis, florid papillomatosis of the nipple, subareolar duct papillomatosis and superficial papillary adenomatosis of the nipple (2,8,12). Since the main feature common to these lesions is adenomatous proliferation in the stroma (small and medium caliber duct proliferation) (1,4), the definition of AN was preferred in the current study.

Although AN are rare and benign entities, the main issue with these lesions is the differential diagnosis with nipple Paget’s disease (clinical and histological diagnosis), DCIS of low-grade, tubular carcinoma, infiltrating syringomatous adenoma and solitary central papilloma subareolar (histological diagnosis) (7).

These lesions are characterized by the presence of two cell populations, an internal layer of cuboidal epithelial cells with an apocrine secretion and an external layer of myoepithelial cells. The presence of a myoepithelial cell layer in neoplastic ducts is considered to be the most important histological observation for distinguishing adenoma from carcinoma. For this reason, the correct immunophenotypic definition, through the use of a panel of specific antibodies for the myoepithelial cells, is always required for the differential diagnosis. Among the frequently used myoepithelial markers are p63, h-caldesmon, calponin 1, α-smooth muscle actin, CK5/6 and CD10 (13,14). The positivity of at least two markers is sufficient for diagnosis. The use of p63 has been largely discussed since it may be extremely useful, particularly for the differential diagnosis with DCIS. In this lesion, the expression of p63 is lost or may appear discontinuous (15). The CK5/6, in addition to myoepithelial cells, is also present within the intraductal epithelial proliferation lesion. In the case of differential diagnosis with atypical ductal hyperplasia and DCIS, positivity for CK5/6 within the ducts is lost (14).

Cytological examination may be performed for diagnosis, but the complete excision of the lesion and examination of FFPE serial sections remains the gold standard for diagnosis. Although the lesion is almost always unilateral, bilateral cases (16,17) and association of AN with malignant breast carcinoma (18–21) have been previously described. With regard to the probability of a tumor developing from these lesions, no reliable data has been identified in the previous literature (22,23).

To date, few case studies have analyzed the numerous individual case reports for AN. A previous case series of 15 cases was described in 1985 by Brownstein *et al* (12). Subsequently, the largest case series was presented in 1986 by Rosen and Caicco with 42 selected cases of AN (8). Finally, a case series of 18 AN cases in the Chinese population was described (9). No previous studies have analyzed the incidence of this lesion in Italy. Since 2002, only single case reports have been presented (2,10).

In the present study, a case series of 13 patients was selected from the National Cancer Institute of Fondazione Pascale Hospital database. This was collected within ten years and represented a female population from the Campania region of Southern Italy. The range of ages of the patients recruited in the study corresponds with that described in the previous literature and the mean age was ~38 years. In addition, the growth pattern frequently found was the papillomatosis pattern with a florid papillary hyperplasia of ductal epithelium. All lesions presented were unilateral and not associated with other malignant diseases of the breast. In all analyzed cases, the definition of the immunophenotypic profile was essential for the correct diagnosis.

In conclusion, although AN may be diagnosed preoperatively by cytological examination and core biopsy, complete excision of the lesion and an adequate histological and immunophenotypic analysis is recommended. This is necessary to discriminate the pseudo invasive pattern that often characterizes this lesion from breast cancer precursors and aggressive carcinoma.

## Figures and Tables

**Table I:** Antibody panel for immunohistochemistry analysis.

Antibody	Source	Clone	Dilution
p63	Rabbit polyclonal	Sc-8343	1:200
h-CALD1	Mouse monoclonal	h-cd	1:400
Calponin	Mouse monoclonal	CALP	1:600
α-smooth muscle actin	Mouse monoclonal	1A4	Prediluted
CD10	Mouse monoclonal	56C6	1:50
CK5/6	Mouse monoclonal	D5/16B4	Prediluted
CK8/18	Mouse monoclonal	5D3-R-7-CE	Prediluted

h-CALD1, h-caldesmon 1; CK, cytokeratin.

**Table II:** Clinicopathological features of patients.

Patient	Age, years	Tumor size, cm	Growth pattern	Myoepithelial markers						CK8/18^a^

				p63	CALD1	CALP1	M-actin	CD10	CK5/6	
1	38	0.9×1.2	Papillomatosis pattern	+	+/−	+	+	+	+	+
2	20	0.8×1.1	Papillomatosis pattern	+	+	+	+	+	+	+
3	40	1.6×1.3	Papillomatosis pattern	+	+/−	+	+	+	+	+
4	31	0.7×1.5	papillomatosis pattern	+	+/−	−	+	+	+	+
5	51	1.2×1.3	Mixed prolif. pattern	+	+/−	−	+	+	+	+
6	42	0.7×1.1	Papillomatosis pattern	+	−	+	+	+	+	+
7	37	1.3×0.9	Papillomatosis pattern	+	+/−	+	+	+	+	+
8	37	0.5×1.2	Papillomatosis pattern	+	+/−	+	+	+	+	+
9	31	1.3×1.5	Papillomatosis pattern	+	+	+/−	+	+	+	+
10	44	0.8×1.2	Papillomatosis pattern	+	+/−	+	+	+	+	+
11	44	1.1×1.3	Papillomatosis pattern	+	+	+/−	+	+	+	+
12	42	0.8×1.3	Mixed prolif. pattern	+	+	+/−	+	+	+	+
13	42	1.2×1.2	Adenosis pattern	+	+	−	+	+	+	+

a Epithelial marker.

CALD1, caldesmon 1; CALP1, calponin 1; M-actin, α-smooth muscle actin; CK, cytokeratin; prolif., proliferation.
